# High-Temperature Oxidation Behavior of Cr-Ni-Mo Hot-Work Die Steels

**DOI:** 10.3390/ma15155145

**Published:** 2022-07-25

**Authors:** Yuqi Zhang, Cheng Zhang, Fei Li, Zhou Wang, Xiaodong Wang, Changji Wang, Cheng Zhang, Jinfeng Huang, Feng Mao, Chong Chen, Tao Jiang, Shizhong Wei, Mei Xiong, Jinmeng Hu

**Affiliations:** 1 School of Materials Science and Engineering, Henan University of Science and Technology, Luoyang 471003, China; zhang18438698876@163.com (Y.Z.); li172224@163.com (F.L.); wangzhou29@163.com (Z.W.); nmxdwang@163.com (X.W.); wchj_1989@163.com (C.W.); maofeng718@163.com (F.M.); chenchong8812@163.com (C.C.); tedivy@163.com (T.J.); xiongmei_1327@163.com (M.X.); 15638265067@163.com (J.H.); 2 National Joint Engineering Research Center for Abrasion Control and Molding of Metal Materials, Henan University of Science and Technology, Luoyang 471003, China; 3 State Key Laboratory for Advanced Metals and Materials, University of Science and Technology Beijing, No. 30, Xueyuan Road, Beijing 100083, China; zhangcheng@ustb.edu.cn (C.Z.); huangjf@ustb.edu.cn (J.H.)

**Keywords:** oxidation, steels, microstructure, grain boundaries

## Abstract

The oxidation of 3Cr3Mo2NiW and 3CrNi3Mo steels was studied at 600 °C in air, and the test results suggest that the parabolic rate law fitted the oxidation kinetics of both steels. The microstructure, morphology, structure, and phase composition of the oxide film cross-sectional layers of the two Cr-Ni-Mo hot-work die steels were analyzed using scanning electron microscopy (SEM), energy-dispersive spectrometry (EDS), and X-ray diffraction (XRD). The influences of Cr, Ni, and Mo on the high-temperature oxidation resistance of the two Cr-Ni-Mo hot-work die steels are discussed, and the oxidation mechanism is summarized. Heat-treated samples were analyzed using electron backscattered diffraction (EBSD) to obtain inverse pole figures (IPFs) and average sample grain sizes, and the percentages of twin grain boundaries (TGBs) (θ = 60°) were also measured. After heat treatment, recrystallization was observed in both steels with a large portion of twin grain boundaries. After 10 h of oxidation, the dense chromium-rich oxide layer that formed in the inner oxide layer of 3Cr3Mo2NiW steel effectively prevented the continuation of oxidation. The inner oxide layer in 3CrNi3Mo steel formed an adhesion layer with a network structure composed mainly of Ni- and Cr-rich spinel oxide, without forming a barrier to prevent oxidation.

## 1. Introduction

Hot-work die steel is widely used in hot forging, pressure hardening, and die casting, where dies are subjected to high temperatures and mechanical loads and are prone to various forms of failure [1,2,3]. In these applications, high-temperature oxidative wear is an important failure mechanism for molds [4,5,6]. The oxide layer produced during high-temperature oxidation gradually changes to a dense enamel layer during high-temperature and mechanical compaction, which was first reported by Stott [7]. However, the organization of the protective oxidized enamel layer produced during high-temperature wear can only be observed after high-temperature wear, and its growth mechanism cannot be explored. For example, some oxide layers flake off during wear, thus nullifying their real impact; therefore, it is necessary to research the resistance of hot-work tool steels to high-temperature oxidation.

Steel oxidation follows the parabolic oxidation regime, and the generated oxide skin possesses a three-layer structure: an outer layer, a transition layer, and an inner layer [8]. In reality, the high-temperature oxidation of steel is much more complex, mainly owing to the complex phases produced by its many alloying elements during oxidation. It is generally accepted that Cr_2_O_3_, SiO_2_, and Al_2_O_3_ are the most protective oxides [9,10]. Moreover, the long-term oxidation resistance of superalloys depends on the compactness, adhesion, and slow growth rate of the oxide layer [11]. During high-temperature oxidation, the high contents of elements in the alloy are essentially oxidized, and the amounts of various oxides in the layer are approximately proportional to the concentrations of elements in the alloy [12].

The element Cr gives the steel high oxidation resistance, which is mainly attributed to the Cr_2_O_3_ generated during oxidation, which has a very low diffusion coefficient for oxygen and metal, and therefore, it is a barrier against further oxidation. During the formation of chromium oxide, the oxide skin has two layers: the inner layer is formed by Cr_2_O_3_, which is dense and adheres to the matrix, and the outer layer is mostly composed of spinel-type oxide MnCr_2_O_4_ [13,14]. X. Jin et al. studied the effect of Mn on the mechanical properties and high-temperature oxidation of 9Cr2WVTa steel. When the content of Mn is between 0.04–0.93 wt.%, the high-temperature oxidation resistance of the alloy is significantly improved with the increase in Mn content [15]. Mo can improve the oxidation resistance of the steel for 10 h below 900 °C; if above 900 °C, the high molybdenum content tends to segregate and form intermetallic compounds, reducing the oxidation resistance and making it crack during hot working [16]. The beneficial effect of Mo on the oxidation resistance of steels has been attributed to several factors, such as the enrichment of Cr and Mo in the oxide layer [17], stabilization of the passive film [18], thickening of the passive film [19], synergistic interaction of Mo ions with other oxides of the passive film [20,21,22], and elimination of active surface sites through the formation of Mo oxides [23]. The addition of Ni improves the high-temperature oxidation resistance of high-strength low-carbon steels [24]. In this study, the static oxidation of hot-work die steel at 600 °C was investigated, and the results can help analyze the enamel layer produced during high-temperature oxidative wear.

## 2. Material and Methods

The chemical composition of hot-work die steel based on composition analysis is listed in Table 1. The heat treatment processes for the two test steels were as follows:

Heat Treatment 1 (3Cr3Mo2NiW): annealed at 870 °C for 2 h, quenched at 980 °C for 1 h, water-cooled, and tempered at 680 °C for 2 h.
Heat Treatment 2 (3CrNi3Mo): annealed at 880 °C for 1 h, quenched at 870 °C for 2 h, water-cooled, and tempered at 680 °C for 2 h.

**Table 1:** Chemical composition of the test steels (mass fraction, %).

Steel	C	Si	Mn	Cr	Ni	Mo	W	P	S	Fe
3Cr3Mo2NiW	0.30	0.02	0.15	3.16	0.50	1.89	0.30	<0.015	<0.015	Bal.
3CrNi3Mo	0.30	0.20	0.20	0.90	2.84	0.20	-	<0.015	<0.015	Bal.

Heat-treated samples were cut to sizes (15 mm × 15 mm × 5 mm and 20 mm × 15 mm × 5 mm) using electrical discharge machining. The small-size specimens were used for scanning electron microscopy (JSM-IT800, JEOL Companies, Tokyo, Japan) observation, and the large-size specimens were used for thermogravimetric analysis and X-ray diffraction (Brux-D8, Bruker AXS Companies, Karlsruhe, Germany) in the physical phase analysis of the oxide layer. This was conducted to prevent the destruction of the oxide layer during secondary processing. The cut specimens were sandpapered and had their edges chamfered. The ground specimens and porcelain boats were ultrasonically cleaned and dried to prevent the interference of moisture in the thermogravimetric analysis of the specimens. The sample was weighed using a five-digit calibration balance until the mass difference between two consecutive readings for the same specimen was less than 0.5 mg. The specimens were put in the furnace at 600 °C, and all oxidations were performed in air. The heating rate of the resistance furnace was set to 30 °C/min. The thermally exposed specimens were pulled out of the furnace and cooled in air after thermal exposure times of 1, 3, 7, or 10 h. 

After oxidation, three specimens of each material at each oxidation time were weighed three times, and the average value for each sample was recorded. Scanning electron microscopy with backscattered electrons (BSE) was used to examine the as-received microstructures and oxide films. Compositional information was gathered using energy-dispersive X-ray spectroscopy (UltimMax40, OXFORD INSTRUMENTS Companies, Oxford, UK). The cross-sectional planes of the EBSD test samples were ion-polished with a cross-section polisher (IB-19530CP, JEOL Companies, Tokyo, Japan) at 7 kV for over 20 min. Electron backscatter diffraction (Oxford C-nano, OXFORD INSTRUMENTS Companies, Oxford, UK) was conducted using step sizes and scanning areas of 0.18 μm and 112 μm by 84 μm for heat-treated samples to obtain inverse pole figures (IPFs) and average sample grain sizes. The percentages of twin grain boundaries (TGBs) (θ = 60°) were also measured. The microstructures of the oxide surface layers of the two Cr-Ni-Mo hot-work die steels were also investigated by XRD. The XRD patterns were obtained using a Brux-D8 diffractometer with Cu Kα radiation at 40 kV and 40 mA. The scanning speed was 6°/min, and 2θ was scanned from 20° to 80°.

## 3. Results and Discussion

### 3.1. Initial Microstructure

**Figure 1:**
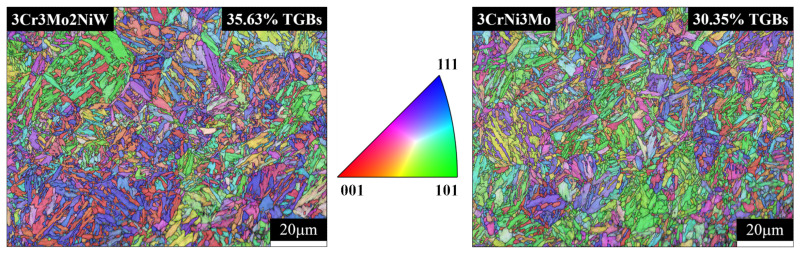
IPFs of two Cr-Ni-Mo hot-work die steels after heat treatment.

Figure 1 shows the patterns of IPFs produced after heat treatment of the samples. The collected IPF maps indicate that the microstructure of the heat-treated hot-work die steel contains recrystallization and equiaxed grains. According to the special grain boundary statistics, the fraction of twin grain boundaries (TGBs) (θ = 60°) in 3Cr3Mo2NiW steel is larger than that in 3CrNi3Mo steel. According to the grain size statistics, the average grain area in the matrix organization of 3Cr3Mo2NiW steel and 3CrNi3Mo steel is about 3.09 μm^2^ and 4.64 μm^2^, respectively. The effect of grain size on the high-temperature oxidation resistance of the alloy is related to the initial Cr content of Cr steels. For low-Cr steels (less than 2.25 wt% Cr), an increase in the grain size improves the oxidation resistance, while steels with high Cr content (18 wt% Cr) can form a thin and protective chromia scale on the surface more easily at a finer grain size [25]. In addition, the higher the twin density, the faster the diffusion path of Cr and the denser the Cr_2_O_3_ protective layer formed [26].

### 3.2. Weight Gain

**Figure 2:**
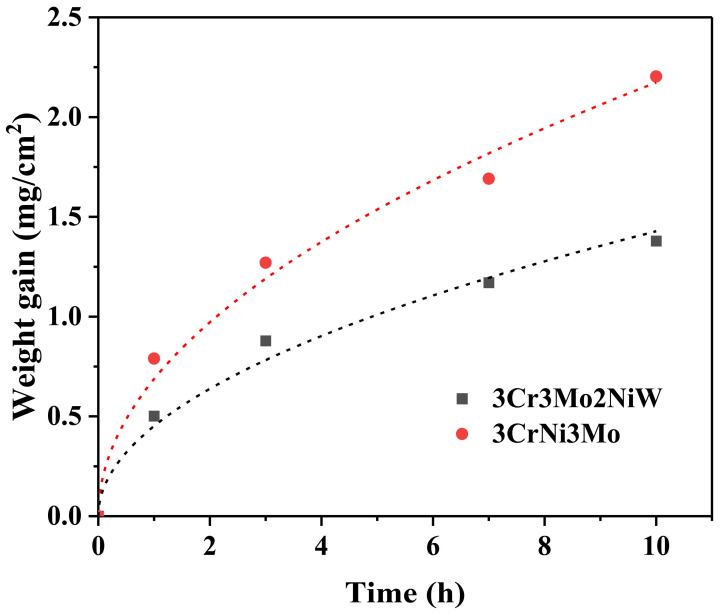
Weight gain as a function of exposure time for the oxidation of two Cr-Ni-Mo hot-work die steels at 600 °C. The lines show the fit to cubic oxidation kinetics.

**Figure 3:**
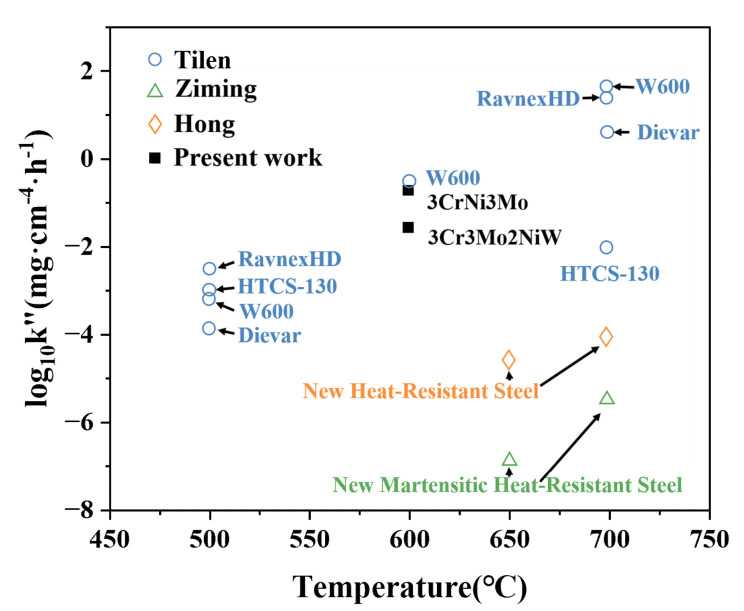
Parabolic rate constants *k”* of oxidation of two hot-work die steels at 600 °C. The other *k”* values of hot-work steels were reported by Tilen [28,29], Ziming [30], and Hong [31].

The parabolic rate law was assumed for the sake of simplicity, and the approximate parabolic rate constants *k*″ (oxidation constant) of the two test steels were calculated from Figure 2 using the equation:

Δ*W*^2^ = *k*″*t* (1)

where Δ*W* is the weight gain per unit area (mg·cm^−2^), and *t* is the oxidation time (h) [27]. The derived *k*″ values are shown in Figure 3 with those from previous studies [28,29,30,31]. As shown in Figure 3, the oxidation constant *k*″ gradually increases as the oxidation temperature rises, indicating a gradual decrease in oxidation resistance with increasing temperature. The two new heat-resistant steels have improved oxidation resistance due to the addition of other elements. This method may also be a means of further improving the oxidation resistance of the two Cr-Ni-Mo hot-work die steels.

The air oxidation kinetics for the two Cr-Ni-Mo hot-work die steels at 600 °C are plotted in Figure 2, and the curves generally represent mean values of the three samples with a standard deviation within ±9%. The oxidation kinetic curves of the two test steels conform to the parabolic equation. This suggests that the thickening of the oxide film leads to a decrease in the metal activity gradient in the oxide film, which in turn leads to a decrease in the ion flux and reaction rate. In Table 2, the correlation coefficients of the fitted equations for the oxidation kinetics of the two steels are in good agreement, ranging between 0.98765 and 0.98816. The growth rate of the oxide layer in 3Cr3Mo2NiW and 3CrNi3Mo steels was 1.39 and 2.20 g·m^−2^·h^−1^, respectively. Thus, both 3Cr3Mo2NiW and 3CrNi3Mo steels exhibit sub-oxidation resistance levels. These results correspond to the thickness of the oxide layer in BSE, as shown in Table 3. Overall, the oxidation of both test steels can be divided into two stages. The first is the rapid formation of the oxide layer, in which the matrix has not yet generated an oxide layer and is in direct contact with air, forming a large amount of oxide of Fe. In the second stage, the dense Cr-containing oxide layer generated on the substrate surface prevents contact between the substrate and oxygen, effectively limiting the diffusion of substrate elements and reducing the rate of high-temperature oxidation.

### 3.3. Phase Characterization of Oxide Film

**Figure 4:**
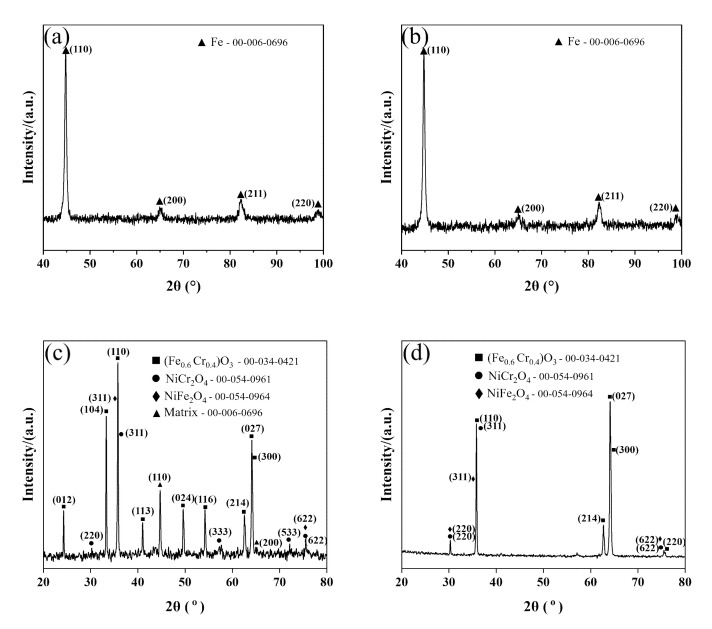
(**a**,**b**) XRD patterns of unoxidized 3Cr3Mo2NiW and 3CrNi3Mo steel; (**c**,**d**) XRD patterns of 3Cr3Mo2NiW and 3CrNi3Mo steels after 10 h of oxidation.

In order to investigate the oxidation products of the two Cr-Ni-Mo hot-work die steels at 600 °C, the two steels were analyzed using X-ray diffraction. As shown in Figure 4, the comparison of the physical phases of the oxide layers of the two test steels shows that 3Cr3Mo2NiW steel contains more Cr-containing oxides, and the 3CrNi3Mo steel contains more Ni-containing spinel oxides. Additionally, 3Cr3Mo2NiW steel shows the characteristic peaks of the steel matrix, indicating that severe spalling of the oxidation scale occurred. The two Cr-Ni-Mo hot-work die steels oxidized for 10 h at 600 °C formed oxides, namely, Cr_2_O_3_, Fe_2_O_3_, and Ni-containing spinel structural oxides.

### 3.4. Cross-Sectional Morphology of the Oxide Film

**Figure 5:**
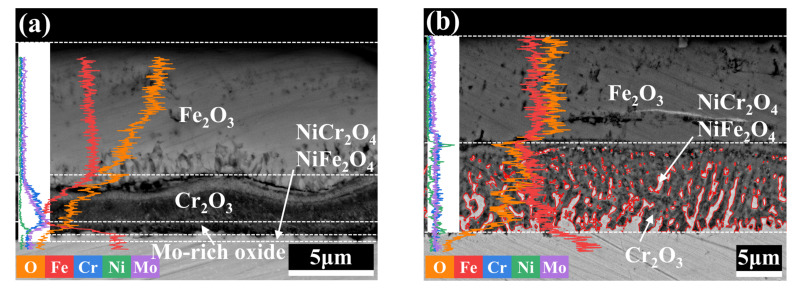
BSE-EDS line profiles of 3Cr3Mo2NiW (**a**) and 3CrNi3Mo (**b**) steel cross-sections.

The formation of the oxide layer is a chemical reaction process, while the thickening of the oxide layer is a combination of diffusion and chemical reaction. The radius of metal ions is significantly smaller than the radius of oxygen ions, which is the reason for the higher migration rate of metal ions, so the oxide layer grows mainly on the outer surface of its growth zone. BSE-EDS of the cross-sections of the samples (Figure 5) shows that the outer oxide layer has a porous morphology. Both internal stresses during oxide growth and volatilization of volatile oxides cause the porous morphology of the oxide layer, and the pores therein are the stress concentration points for oxide crack sprouting [32]. The volatilization of MoO_3_ and the higher silicon content may be the reason for the porous layer in both Cr-Ni-Mo hot-work die steels [33,34]. After high-temperature oxidation, the outer oxide layers of the two Cr-Ni-Mo hot-work die steels are enriched with Fe elements. The internal oxide layer of 3Cr3Mo2NiW steel is rich in Cr, Mo, and Ni elements, which may be the generative zone of Cr2O3, Fe2O3, Mo oxides, and Ni-containing spinel structural oxides. The EDS line profiles of 3Cr3Mo2NiW steel show that the element aggregation in the inner layer of the oxide layer is mainly caused by Ni elements, and its oxide may be mainly Ni-containing spinel structural oxides.

**Figure 6:**
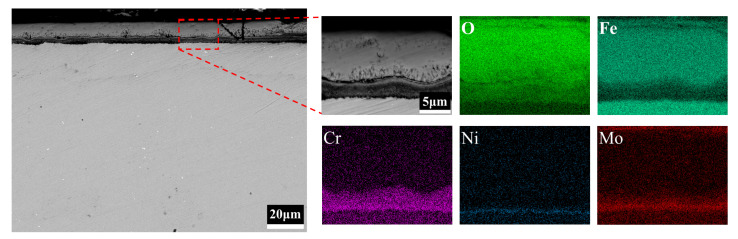
BSE-EDS maps of 3Cr3Mo2NiW steel cross-section.

Figure 6 shows the cross-sectional morphology and elemental distributions of 3Cr3Mo2NiW steel specimens measured by EDS mapping. The Cr-rich oxide layer produced by the oxidation of 3Cr3Mo2NiW steel is wavy, with tiny gaps between both the inner and outer layers in Figure 6 (BSE). This is caused by the presence of high growth compressive stresses in the polycrystalline oxide layer, and the generation of this compressive stress can be explained by the formation of new oxides at the grain boundaries of the chromium oxide films [35]. In Figure 6 (EDS maps), the outer layer of the oxide film of 3Cr3Mo2NiW steel is mainly composed of Fe and O elements, and there are aggregated bands of Cr, Ni, and Mo elements in the inner layer. The selective oxidation of Cr elements to form Cr_2_O_3_ leads to a decrease in the Cr concentration in the surface layer of the steel, resulting in a concentration gradient from the surface to the inside. Combined with the XRD patterns of 3Cr3Mo2NiW steel in Figure 4, it can be concluded that the outer layer of the oxide film is mainly Fe_2_O_3_, and the inner layer is mainly Cr_2_O_3_, NiCr_2_O_4_, and NiFe_2_O_4_. Compared with 3CrNi3Mo steel, the relatively dense Cr-rich oxide layer generated in the inner layer of the oxide film of 3Cr3Mo2NiW steel forms a barrier, which prevents further oxidation of the substrate and plays a positive role in oxidation resistance. 

**Figure 7:**
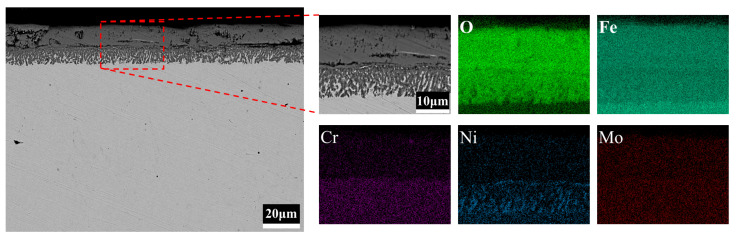
BSE-EDS maps of 3CrNi3Mo steel cross-section.

Figure 7 shows the cross-sectional morphology and elemental distributions of 3CrNi3Mo steel specimens measured by BSE-EDS. The inner layer of the oxide film of 3CrNi3Mo steel formed an oxide with a reticulated structure, as shown in Figure 7 (BSE). These reticulated oxides are highly adhesive and provide a tight connection between the oxide layer and the substrate. In Figure 7 (EDS), the outer oxide film of 3CrNi3Mo steel is mainly composed of Fe and O elements, and there are reticulated aggregation areas of Ni elements in the inner layer. Combined with the XRD pattern of 3CrNi3Mo steel in Figure 4, it can be seen that the reticular aggregation zone of Ni elements is Ni-rich reticular spinel oxide. However, the aggregation of Cr and Mo elements in the inner layer of the oxide film of 3CrNi3Mo steel is weaker than that in 3Cr3Mo2NiW steel. This is mainly caused by the different concentrations of elements in the two Cr-Ni-Mo hot-work die steels [12]. The formation of Ni-rich spinel structured oxides in the internal oxide layer of 3CrNi3Mo steel hinders the formation of the passivation film and therefore fails to form a barrier against further oxidation of the matrix.

### 3.5. Oxidation Mechanism

The formation of the oxide layer is a chemical reaction process, and the thickening of the oxide layer is a combined process of diffusion and chemical reaction. The radius of metal ions is significantly smaller than the radius of oxygen ions, which is the reason for the higher migration rate of metal ions, so the oxide layer grows mainly on the outer surface of its growth zone. In the cross-sectional scanning images of the two Cr-Ni-Mo hot-work die steels after oxidation for 10 h at 600 °C, an oxide layer is present in all samples. The average thicknesses of the oxide layers of 3Cr3Mo2NiW and 3CrNi3Mo steels after oxidation at 600 °C for 10 h are about 12.55 and 19.89 μm, as shown in Table 3. According to the analysis of Figure 3, Figure 4 and Figure 5, mainly the oxides Fe_2_O_3_ and Cr_2_O_3_ are present in the oxide layer. This is mainly due to the different oxidation activities of the elements, and the elements with high activity are easily and preferentially oxidized, i.e., selective oxidation [36]. The oxidation activity of the elements, i.e., their affinity for oxygen, is related to their free energy of formation of oxides. At the same time, according to Equations (2) and (3),

B + νO = BO_ν_ (2)

G′ = H′ − TS′(3)

where BO_ν_ is a solute metal oxide, G′ is Gibbs free energy, H′ is the enthalpy change, T is the temperature, and S′ is the entropy change.

**Figure 8:**
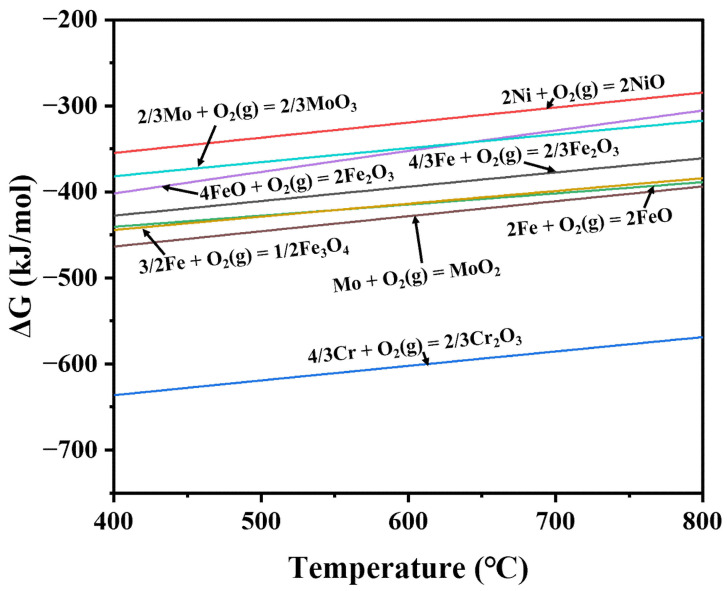
Relationship between the free energy produced by the oxide and the temperature.

Internal oxidation occurs when the free energy G’ of the oxidation reaction is below 0 and relatively low. The chromium in the matrix oxidizes first, and then the iron and nickel begin to oxidize, as shown by the calculated oxidation Gibbs free energies for the alloying elements (Table 4 and Figure 8). Although Cr is preferentially oxidized to produce Cr_2_O_3_, the amount of Cr in the matrix is not sufficient to support the formation of a continuous single protective oxide layer. The reaction of NiO with Cr_2_O_3_ produces the spinel oxide NiCr_2_O_4_, which also has some protective properties. The spinel oxides embedded in the oxide scale also break the continuity of the chrome oxide scale and lead to the “breakaway” for the diffusion of oxygen and metallic ions, resulting in the catastrophic oxidation of 3CrNi3Mo steel (Figure 5b). When the oxide film produced by internal oxidation reaches a certain thickness, the transport mechanism of reactants through the oxide film becomes an important part of the high-temperature oxidation mechanism. As the oxide film becomes thicker, the concentration of solute required for external oxidation increases, which is why the oxidation process follows a parabolic law.

**Figure 9:**
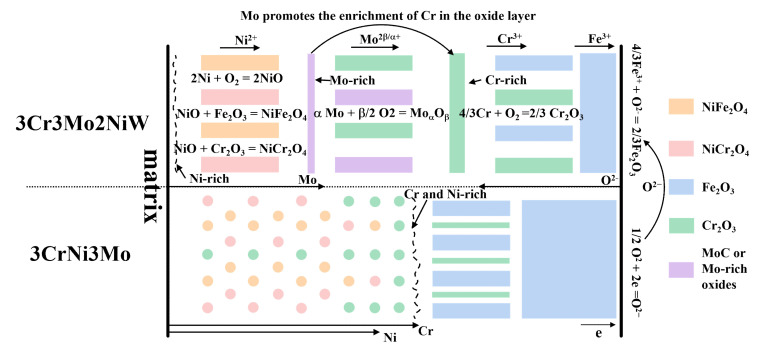
Schematic diagram elucidating the oxidation mechanism of two Cr-Ni-Mo hot-work die steels at 600 °C.

In summary, the reasons for the good oxidation resistance of hot-work die steel are twofold (Figure 9). First, the enrichment of Mo in the oxide layer hinders the outward diffusion of Fe, while the inward diffusion of O_2_ is also inhibited, stabilizing the passivation film and increasing the thickness of the passivation film. Second, a dense protective layer of Cr_2_O_3_ is formed in the oxide layer.

## 4. Conclusions

1. At 600 °C, the two Cr-Ni-Mo hot-work die steels exhibit sub-oxidation resistance levels and conform to the Δ*W*^2^ = *k*″*t* oxidation mode. The oxidation resistance of the test steels at high temperature is closely related to the Cr and Mo contents.
2. The reason for the better oxidation resistance of 3Cr3Mo2NiW steel is mainly its higher content of Cr and Mo. The Cr-rich oxide layers generated during oxidation act as barriers against further oxidation and inhibit the continued oxidation of the substrate. Mo can promote the formation of Cr-rich oxide layers. In addition, the higher twin density and grain refinement may also be reasons for its good oxidation resistance.
3. Although the oxide layer of 3CrNi3Mo steel is thick, the inner part of its oxide layer forms a large number of Ni-containing spinel structural oxides, and the larger thickness of the inner layer and the mesh structure improve the adhesion of the oxide layer.
4. In the process of high-temperature oxidation of the two Cr-Ni-Mo hot-work die steels, the interface reaction initially dominates, and the diffusion process gradually becomes the dominant oxidation factor with the thickening of the oxide film.

## Figures and Tables

**Table 2:** Oxidation kinetic equations of two Cr-Ni-Mo hot-work die steels.

Steel	Oxidation Constant (*k*″)	Oxidation Kinetic Equation	Correlation Coefficient (R^2^)
3Cr3Mo2NiW	0.20 ± 0.01	Δ*W*^2^ = 0.20 *t*	0.99
3CrNi3Mo	0.47 ± 0.03	Δ*W*^2^ = 0.47 *t*	0.99

**Table 3:** Average thickness of oxide layer for two Cr-Ni-Mo hot-work die steels.

Steel	Average Thickness of Oxide Layer (μm)
3Cr3Mo2NiW	12.55
3CrNi3Mo	19.89

**Table 4:** Reactions for the oxidation of Fe, Ni, and Cr in Cr-Ni-Mo hot-work die steels at 600 °C. The values of the standard Gibbs free energy changes of these reactions ΔrG° were calculated according to the thermodynamic data from HSC Chemistry version 6.0 database (#).

Metals	Actions	ΔrG° (kJ/mol)
Fe	4/3 Fe + O_2_ (g) = 2/3 Fe_2_O_3_ (1)	−393.846 ^#^
Ni	2Ni (s) + O_2_ (g) = 2NiO (s) (2)	−319.296 ^#^
Cr	4/3 Cr + O_2_ (g) = 2/3 Cr_2_O_3_ (3)	−602.199 ^#^
	NiO (s) + Fe_2_O_3_ (s) = NiFe_2_O_4_ (s) (4)	−23.569 ^#^
	NiO (s) + Cr_2_O_3_ (s) = NiCr_2_O_4_ (s) (5)	−6.635 ^#^

## Data Availability

All data are available within the manuscript.

## References

[1] Boher C., Le Roux S., Penazzi L., Dessain C. (2012). Experimental investigation of the tribological behavior and wear mechanisms of tool steel grades in hot stamping of a high-strength boron steel. Wear.

[2] Terek P., Kovačević L., Miletić A., Panjan P., Baloš S., Škorić B., Kakas D. (2016). Effects of die core treatments and surface finishes on the sticking and galling tendency of Al–Si alloy casting during ejection. Wear.

[3] Wei M., Wang F., Wang S., Cui X. (2009). Comparative research on the elevated-temperature wear resistance of a cast hot-working die steel. Mater. Des..

[4] Cui X., Wang S., Wang F., Chen K. (2008). Research on oxidation wear mechanism of the cast steels. Wear.

[5] Pellizzari M., Cescato D., Flora M.G.D. (2009). Hot friction and wear behaviour of high speed steel and high chromium iron for rolls. Wear.

[6] Hernandez S., Hardell J., Winkelmann H., Ripoll M.R., Prakash B. (2015). Influence of temperature on abrasive wear of boron steel and hot forming tool steels. Wear.

[7] Stott F., Lin D., Wood G. (1973). The structure and mechanism of formation of the ‘GLAZE’ oxide layers produced on nickel-based alloys during wear at high temperatures. Corros. Sci..

[8] Davies M.H., Simnad M.T., Birchenall C.E. (1951). On the Mechanism and Kinetics of the Scaling of Iron. JOM J. Miner. Met. Mater. Soc..

[9] Huang J., Fang H., Fu X., Huang F., Wan H., Zhang Q., Deng S., Zu J. (2000). High-Temperature Oxidation Behavior and Mechanism of a New Type of Wrought Ni–Fe–Cr–Al Superalloy up to 1300 °C. Oxid. Met..

[10] Col A., Parry V., Pascal C. (2017). Oxidation of a Fe–18Cr–8Ni austenitic stainless steel at 850 °C in O_2_: Microstructure evolution during breakaway oxidation. Corros. Sci..

[11] Li K., Zeng Y., Luo J.-L. (2021). Corrosion of SS310 and Alloy 740 in high temperature supercritical CO_2_ with impurities H_2_O and O_2_. Corros. Sci..

[12] Pint B.A., Distefano J.R., Wright I.G. (2006). Oxidation resistance: One barrier to moving beyond Ni-base superalloys. Mater. Sci. Eng. A.

[13] Huntz A.M., Reckmann A., Haut C., Sévérac C., Herbst M., Resende F.C.T., Sabioni A.C.S. (2007). Oxidation of AISI 304 and AISI 439 stainless steels. Mater. Sci. Eng. A.

[14] Karimi N., Riffard F., Rabaste F., Perrier S., Cueff R., Issartel C., Buscail H. (2008). Characterization of the oxides formed at 1000°C on the AISI 304 stainless steel by X-ray diffraction and infrared spectroscopy. Appl. Surf. Sci..

[15] Jin X., Chen S., Rong L. (2017). Effects of Mn on the mechanical properties and high temperature oxidation of 9Cr2WVTa steel. J. Nucl. Mater..

[16] Li H., Zhang B., Jiang Z., Zhang S., Feng H., Han P., Dong N., Zhang W., Li G., Fan G. (2016). A new insight into high-temperature oxidation mechanism of super-austenitic stainless steel S32654 in air. J. Alloys Compd..

[17] Doh S.J., Je J.H., Kim J.S., Kim K.Y., Kim H.S., Lee Y.D., Lee J.M., Hwu Y. (2003). Influence of Cr and Mo on the passivation of stainless steel 430 (18Cr) and 444 (18Cr-2Mo): In situ xanes study. Nucl. Instrum. Methods Phys. Res..

[18] Li D.G., Wang J.D., Chen D.R., Liang P. (2015). Influence of Molybdenum on Tribo-Corrosion Behavior of 316L Stainless Steel in Artificial Saliva. J. Bio- Tribo-Corros..

[19] Schultze J.W., Lohrengel M.M., Ross D. (1983). Nucleation and growth of anodic oxide films. Electrochim. Acta.

[20] Yun D.W., Seo H.S., Jun J.H., Lee J.M., Kim K.Y. (2012). Molybdenum effect on oxidation resistance and electric conduction of ferritic stainless steel for SOFC interconnect. Int. J. Hydrogen Energy.

[21] Buscail H., El Messki S., Riffard F., Perrier S., Cueff R., Issartel C. (2008). Role of molybdenum on the AISI 316L oxidation at 900 °C. J. Mater. Sci..

[22] Wu Q., Liu Y., Zhang Z., Qi Y., Zhang C., Zheng H., Xu Y. (2021). Oxidation behavior and high-temperature tensile properties of Fe-9Cr-(Mo, Mo/Ni) alloys. Corros. Sci..

[23] Teramoto K.H.A. (1979). An X-ray photo-electron spectroscopic study on the role of molybdenum in increasing the corrosion resistance of ferritic stainless steels in HCl. Corros. Sci..

[24] Liu Y.-Z., Yang C.-F., Chai F., Pan T., Su H. (2014). High Temperature Oxidation Resistance of 9Ni Steel. J. Iron Steel Res. Int..

[25] Trindade V., Christ H.-J., Krupp U. (2010). Grain-Size Effects on the High-Temperature Oxidation Behaviour of Chromium Steels. Oxid. Met..

[26] Ma W., Luo H., Yang X. (2020). The Effects of Grain Size and Twins Density on High Temperature Oxidation Behavior of Nickel-Based Superalloy GH738. Materials.

[27] Zhang L.L., Yan S., Jiang S. (2015). Hard X-ray micro-focusing beamline at SSRF. Nucl. Sci. Tech..

[28] Balaško T., Burja J., Medved J. (2018). High-temperature oxidation of four hot-work tool steels. Mater. Tehnol..

[29] Balaško T., Vončina M., Burja J., Batič B.Š., Medved J. (2022). High-Temperature Oxidation Behavior of Tool Steel with Increased Thermal Conductivity. Oxid. Met..

[30] Bao Z., Han R., Zhu Y., Li H., Li N., Tang M., Zhang H., Zhao C. (2022). High Temperature Oxidation Behavior of New Martensitic Heat-Resistant Steel. Mater. Sci..

[31] Li H., Zhao C., Yan T., Ding C., Zhang H., Jiang F. (2019). Properties of High Temperature Oxidation of Heat-resistant Steel with Aluminium and Copper. Mater. Sci..

[32] Pang H.T., Reed P.A.S. (2007). Microstructure effects on high temperature fatigue crack initiation and short crack growth in turbine disc nickel-base superalloy Udimet 720Li. Mater. Sci. Eng. A.

[33] Taniguchi S., Yamamoto K., Megumi D., Shibata T. (2001). Characteristics of scale/substrate interface area of Si-containing low-carbon steels at high temperatures. Mater. Sci. Eng. A-Struct. Mater. Prop. Microstruct. Process..

[34] Li W., Fan J., Fan Y., Xiao L., Cheng H. (2018). MoSi2/(Mo, Ti)Si-2 dual-phase composite coating for oxidation protection of molybdenum alloy. J. Alloys Compd..

[35] Hirbod M. (1989). The Influence of Fine Structure, Morphology and Composition of Alloy and Oxide on the Growth of Cr_2_O_3_ Scales.

[36] Wang J., He Q., Liu G., Zhang Q., Liu G., Huang Z., Zhu X., Fu Y. (2021). High-Temperature Oxidation Behavior of AlTiNiCuCox High-Entropy Alloys. Materials.

